# 89590 A multi-theoretical analysis of the design, implementation and outcomes of The Greater Rochester LARC Initiative to prevent unintended teen pregnancy

**DOI:** 10.1017/cts.2021.546

**Published:** 2021-03-30

**Authors:** C. Andrew Aligne, Jessica L. VanScott, Reza Yousefi-Nooraie, Katherine Blumoff Greenberg, Rachael H. Phelps

**Affiliations:** 1 The Hoekelman Center, Department of Pediatrics, University of Rochester School of Medicine and Dentistry; 2 Department of Public Health Sciences, University of Rochester School of Medicine and Dentistry; 3 Departments of Pediatrics and Obstetrics/Gynecology, University of Rochester School of Medicine and Dentistry

## Abstract

ABSTRACT IMPACT: This study provides insights on how to replicate a successful initiative for preventing unintended teen pregnancy. OBJECTIVES/GOALS: Reducing unintended teen pregnancy is a national health priority, and a recommended strategy is to increase awareness and availability of long-acting reversible contraception (LARC). The Rochester LARC Initiative did this, and teen LARC use rose from 4% to 24%. The goal of this study is to determine key elements for replicating the intervention. METHODS/STUDY POPULATION: Our initiative used an innovative approach we call ‘community detailing’ to deliver education about LARC to adults working with teens. We analyzed the intervention goals, design components, implementation strategies, and public health outcomes. Our analysis was informed by the CDC model for Promoting Science-Based Approaches to Teen Pregnancy Prevention Using Getting to Outcomes (PSBA-GTO), Diffusion of Innovations, and RE-AIM framework for implementation outcomes. We compared our model with characteristics of LARC-promotion efforts, as well as successful health education campaigns. We tabulated the components of our intervention across theoretical domains, aiming to determine essential elements of effective design, adaptation, and dissemination & implementation. RESULTS/ANTICIPATED RESULTS: The initiative incorporated multiple components common to successful health education programs: measurable behavior-change outcomes; formative research before roll-out; tailored communications for different audiences; speakers who were credible, knowledgeable and skilled communicators; content that was new to recipients and essential for decreasing barriers to desired behaviors. It included elements of successful LARC promotion/teen pregnancy prevention programs, such as organizing information by effectiveness of methods and using youth-empowering messaging. It differed from other successful programs by offering discussions to adults who work with teens in both medical and community settings. This analysis also highlights unintended positive ripple effects. DISCUSSION/SIGNIFICANCE OF FINDINGS: These results establish how community detailing is effective for disseminating actionable information about the safety, efficacy and availability of LARC. These insights could inform other prevention initiatives. An anticipated practical product of this study will be a user-friendly manual for replicating the LARC Initiative in other locations.

